# 1139. Oral Antivirals Against SARS-CoV-2: a New Valid Strategy Against COVID-19

**DOI:** 10.1093/ofid/ofac492.977

**Published:** 2022-12-15

**Authors:** Neva Braccialarghe, Rosario Alessandro Cavasio, Drieda Zaçe, Ilaria Spalliera, Luigi Coppola, Iannetta Marco, Loredana Sarmati, Massimo Andreoni

**Affiliations:** University of Rome Tor Vergata, Rome, Lazio, Italy; University of Rome Tor Vergata, Rome, Lazio, Italy; University of Rome Tor Vergata, Rome, Lazio, Italy; Tor Vergata University of Rome, Rome, Lazio, Italy; Tor Vergata Hospital, Rome, Lazio, Italy; University of Rome Tor Vergata, Rome, Lazio, Italy; Tor Vergata University of Rome, Rome, Lazio, Italy; Tor Vergata University of Rome, Rome, Lazio, Italy

## Abstract

**Background:**

Since the outbreak of COVID-19 pandemic the scientific community efforts have been focused on finding a vaccine and a treatment for infected people. Several antivirals with activity against SARS-CoV-2 were investigated and since the end of 2021 orally administered antivirals have been used in positive patients.

**Methods:**

We conducted a retrospective, single-center study including data from 135 outpatients who resulted positive for SARS-CoV-2 and were selected according to AIFA criteria to receive Molnupiravir (M) or Nirmatrelvir/Ritonavir (N/R) in the Infectious Disease Clinic of the Tor Vergata Hospital from January 2022 to February 2022.

**Results:**

Our cohort included 135 patients with a median age of 71 years (IQR 56,5 – 80,5), 51% were male, 91% received M and 9% received N/R. The median time of antiviral administration from symptoms onset was of 3,4 days. 75% of patients were vaccinated with booster dose, 19% were vaccinated with two doses and 7% were unvaccinated. The most frequent criteria of eligibility are summarized in Figure 1. Only 2 patients were hospitalized receiving oxygen support, 1 patient died, 84% of patients did not need hospitalization and 14% of the enrolled subjects were lost at follow up. Time of negativization at the nasopharyngeal swab (NPS) had a negative correlation to the value of anti-Spike (r=-0.29; p=0.01).

The difference between cycle threshold (Ct) value of NPS at T7 and Ct value at the first positive NPS (T0) was higher among not hospitalized (NH) vs hospitalized (H) patients, for Gene E [12.1 (SD 5.8) vs -1.7 (SD 10.8); p=0.02], Gene N [12.3 (SD 10.8) vs -0.9 (SD 11.6); p=0.01] and Gene RdRp [11.8 (SD 5.3) vs -1.5 (SD 11.1); p=0.01]. Anti-Spike were higher among NH vs H patients [1714 (SD 1044.7) vs [43 (SD 60.3); p=0.028].

Delta Ct E, delta Ct N and delta Ct RdRp were significantly higher in patients without neoplastic disease (p=0.04, p=0.02, p=0.01, respectively) and had a negative correlation with creatinine levels (r=-0.36, p< 0.001; r=-0.3, p=0.03; r=-0.32, p=0.02).

Percentage of eligibility criteria of our patients

**Figure:**
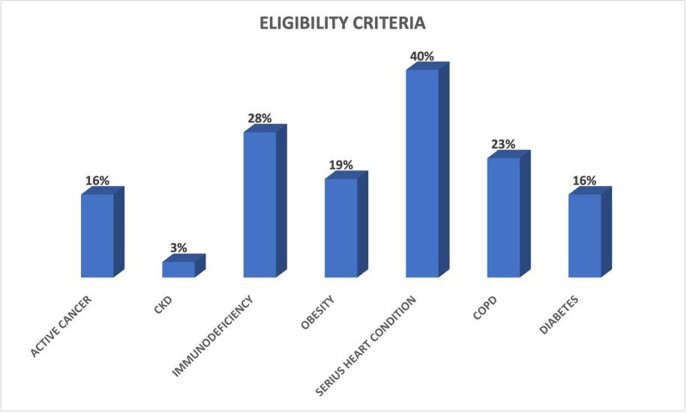

The majority of patients had more than one criterion

**Conclusion:**

Oral antivirals represent a new effective treatment against COVID-19 in selected patients, reducing the time of negativization and the risk of hospitalization and death. Solid tumor or oncohematologic diseases are associated to a slower negativization of NPS.

**Disclosures:**

**All Authors**: No reported disclosures.

